# Genito-Urinary Epithelium1Read at the seventy-sixth annual meeting of the Medical Society of the County of Erie.

**Published:** 1898-04

**Authors:** Thomas B. Carpenter

**Affiliations:** Buffalo, N. Y.


### GENITO-URINARY EPITHELIUM.[1]

By THOMAS B. CARPENTER, M. D., Buffalo, N. Y.

THE later text-books upon urinalysis and diseases of the urinary tract lay far too little stress upon the importance of recognising and determining the exact source of epithelial cells found in the urine. Many observers claim that no reliable inferences can be drawn from cellular elements appearing in the urine,

1. Read at the seventy-sixth annual meeting of the Medical Society of the County of Erie.

either as to the source of the cells or the existence of an abnormal condition, provided the source could be determined. The following lines, quoted from Professor Purdy's recent edition of Practical urinalysis and urinary diagnosis, presents very briefly some opposing views :

> It was formerly believed that the various divisions of the urinary tract possessed their own special form of epithelium and, therefore, the special forms of epithelial cells found in the urine became valuable aids in locating the seat of lesions of the urinary tract. This view is, indeed, still held by a number of prominent observers. More accurate and extensive observations, however, have shown that this can only be depended upon in a very general way. Very often the epithelium claimed to be characteristic of certain divisions of the urinary tract has been found in all its typical peculiarities in a totally different location. This, however, is more likely to be the case in divisions more nearly located to each other. The divergent views upon this point, held even by the ablest and most experienced observers, may be illustrated by the following : Sir William Roberts describes the epithelium shed from the kidney pelvis as that of "very irregular, three-cornered, elongated, rudely circular, etc." Dr. Dickinson has carefully figured the epithelium taken from the bladder and in reply laconically observes : "It will be seen that these varieties of, even to the *et ceteræ*, are equally characteristic of vesical disease."

Dr. Dickinson's reply to Sir William Roberts makes no observation upon variations in size between the cells from the kidney pelvis and the bladder, neither has the exact source of the cells been noted. Variations in size and shape exist, not only between the cells of different parts, but between the cells of the same part depending on the condition at that place, the existence or non-existence of inflammation, and whether the cells are superficial or deeply seated in the lining membrane. Sufficient variations in size and shape can be found to accurately determine the previous location of cells as they occur in the urine. Many times it is difficult or even impossible to definitely locate a lesion from the clinical symptoms in a certain case, and in these cases the information gleaned from a careful observation of the urine, and particularly the cells occurring in it, is of inestimable value. In most cases a more accurate diagnosis of the exact seat and extent of a lesion can be made by this means than from the symptomatology. The cells as they appear *in situ* will be considered first, after which their appearance in the urine.

Beginning at Bowman's capsule, we find, as a rule, the tubular portion of the kidney lined with a single layer of cells. Some observers (Klein) note the finding of an additional layer of cells intimately attached to the membrana propria of the larger collecting tubules. In Bowman's capsule the lining consists of polyhedral, columnar cells, changing to squamous in the adult. Passing through the neck of the capsule to the proximal convoluted tubule, the cells are polyhedral or short columnar and may be angular or club-shaped. It is of interest to note that in some of the lower animals ciliated cells have been found in this situation, but so far as I know nothing of this character has been noted in man. The cells of the proximal convoluted tubule are peculiar in that the nucleus is situated eccentrically in a mass of granular protoplasm, and the remainder of the cell, that part attached to the membrana propria, has a fibrillated appearance, the fibrils running at right angles to the tubular wall. This peculiar structure is common to a greater or lesser degree of all portions of the tubular structure down to the collecting tubules, but most marked in those portions situated in the labyrinth. This peculiarity of structure is supposed to have to do with the separation of the saline constituents of the blood.

Passing downward to the spiral tubule the cells possess practically the same characteristics, but granulation and fibrillation are less marked. Continuing into the descending loop-tube of Henle the cells change to a marked degree. No evidence of fibrillation is apparent, they lose the columnar shape, becoming squamous, less granular, very transparent and possess an oval nucleus. After passing Henle's loop the cells change again to the polyhedral variety, are larger in size than those from the proximal convoluted tubule and show slight indication of fibrillation. In the irregular tubule, between the ascending tube of Henle and the distal convoluted tubule, the cells are polyhedral, pyramidal or short columnar and possess a flat, oval nucleus. The fibrillated appearance is at this point very coarse and well marked. In the first part of the collecting tubule the cells are flat, polyhedral and quite transparent. As we go lower down in the collecting tubule the cells tend toward a battledore shape and are considerably larger than in any portion yet considered. This observation is at variance with some investigators, but certainly true, as careful study of the cells will show.

Descending to the pelvis of kidney, decided changes are noticeable. Instead of a single layer of cells, we find the lining to consist of several layers,—so-called stratified trasitional epithelium,—meaning, of course, that there are two or more layers of cells, developing from the bottom upward and undergoing transitions in shape as the surface is reached. In the lower layers of the renal pelvis the cells are approximately round, about twice the size of a pus corpuscle and usually refractive in appearance, with a well-marked nucleus. As the surface is reached the cells assume a battledore shape characteristic of this locality. This stratified transitional epithelium is common to the remainder of the urinary tract with few exceptions, to be noted later.

According to Klein, "the epithelium of the ureters and bladder is transitional epithelium. It is stratified, the most superficial layer consisting of squamous cells; underneath this a layer of club-shaped cells, between which extend one or more layers of small spindle-shaped cells." I cannot substantiate this statement as a whole, for in my observations the superficial layer of cells consists of the spindle-shape and are comparatively large. The quotation is true for the bladder, but such a great variation in size exists that distinction is easy.

Passing now to the neck of bladder and the prostatic urethra, we find the same transitional, stratified epithelium, but differing greatly in size from that of other locations. The superficial cells of this region are of the circular, squamous variety and are much more refractive than cells from any other region. Along the remainder of the urethra the cells are of the simple, columnar variety, until the fossa navicularis is reached, where again we find cells that are very similar to the prostatic. In this connection it is well to note that after chronic urethral inflammations the columnar cells are replaced by pavement cells (Finger).

In the female urethra stratified, transitional epithelium is also found, except at the orificium externum, where the cells are of the stratified, pavement type, without transitional forms. The superficial layers of the female urethra have been usually described as club-shaped. This I have not noted, always finding them round or polygonal and approximating the male prostatic cells in appearance. In the vagina about the same character of cell is found as in the bladder, differing in the usual larger size of the superficial vaginal cell, also in the fact that the vaginal cells overlap each other when in small shreds, instead of simple contact and cementation, as in the bladder.

It is exceptional for any large amount of epithelium to be thrown off and voided in the urine at one time; it only occurs in the most acute inflammations. Chronic inflammations will in time entirely denude a surface, but regeneration is rapid and it is in extreme cases only that sufficient information cannot be obtained from the epithelium present. Unfortunately, epithelial cells are elastic and change their shape as the part to which attached and after detachment the action of the saline urine still further changes them. Polyhedral and columnar cells are apt to become globular; small, squamous cells will also swell and appear quite different than when observed *in situ*. These points, as well as the condition of the surface from which the cells may come, must be considered. In shock, following severe injuries, with resulting internal congestions, all the cells of the urinary tract may become swollen and distorted. The character, extent and duration of an inflammation will also alter the appearance of the cells. Rapid regeneration will result in distorted forms and old inflammations with sluggish cell formation result in fatty degenerated cells, more or less distorted. Inflammations of an asthenic type produce characteristic transparent cells that will rapidly disintegrate.

Let us now consider from a practical standpoint the epithelial cells as they occur in the urine and note briefly how they may vary from the descriptions already given. The cells from the renal tubules are always round, except those coming from the larger collecting tubules. These cells usually have a process jutting forth from some portion of the periphery and thus tend toward the battledore shape. Variations in size can be noted, depending upon the portion of the kidney from which they come. Those cells from the smaller tubules are usually somewhat smaller than a pus corpuscle and gradations in size are seen, up to those half as large again as a pus corpuscle and coming from the larger tubules. The only cells that approximate the renal in appearance are those from the urethra and its accessory glands. Differentiation is easy because the cells from both places always have characteristic accompaniments: the renal cells by casts and the urethral cells by pus. The appearance of renal cells in normal urine is exceptional.

The superficial cells of the kidney pelvis are of the battledore shape and are seen typically in acute rapid processes where the cells are often shed in scales, the individual cells overlapping like the slates on a roof. In chronic processes the superficial cells may disappear entirely and only the slightly larger round cells of the deeper layers be found in the sediment. Affections of the renal pelvis are of so much importance and difficult of diagnosis that a more extended description of the epithelial accompaniments will be given. As pointed out by Ultzman some years since, pus appears in the urine, both as free separated corpuscles and in the form of clumps, differing in size and appearance, depending upon the source. The shreds, so well known as occurring in gonorrheal urine, represent one form of pus clumping, due either to the rolling up of the secretion as the urine passes it through the urethral canal, or, in some cases, to the tenacious character of the secretion. The same physical condition presents itself in pus from the renal pelvis and due to the same causes. The pus is rolled into clumps as it passes through the ureters, but differing from the urethral and prostatic clumps by the tighter clumping and the smaller size of the clumps. In some instances the clumping is due to packing of corpuscles in the apices of the calices or in large collecting tubules. The purulent plugs or clumps are associated with the characteristic cells of the part from which they come.

Cells from the ureters are not often found in the urine because the ducts are usually not affected in the passage upward of an inflammation. After passage of a small renal calculus the cells may be found in abundance and are of the spindle or double caudate variety.

Cells from the superficial layer of the bladder are of the large squamous type and are found in quantity only in the acute inflammations. In chronic cystitis the cells are not numerous and oftentimes coming from the deeper layers may simulate in shape the cells from nearly all portions of the urinary tract. As a rule the bladder cells that might be mistaken for those from other localities are much larger and the character of accompaniments generally suffice for an accurate localisation. Approaching the neck of bladder, the cells become much smaller, more nearly circular and so refractive in appearance that mistake is not likely to occur. This peculiar refractive appearance is common to three portions of the urinary tract—namely, fossa navicularis, prostate (opposite internal and external sphincter muscles) and convoluted tubules where the tubule joins Bowman's capsule. It is also noticeable in the female urethra near the meatus.

Cells from the male urethra between the prostatic portion and the fossa navicularis are usually round, as appearing in the urine, and somewhat larger than a pus corpuscle. If the urine is fresh and only a short time elapses between the time of voiding and examination, the more nearly the columnar shape is retained.

Cells from the vagina are often a confusing factor in the examination of female urines and are usually present in considerable numbers, even in normal samples. Their size and number, overlapping, if present in shreds, and the absence of pus usually suffices to localise.

325 Jersey Street.

